# Construction of the Armenian Surname List (ASL) for public health research

**DOI:** 10.1186/s12874-023-01848-1

**Published:** 2023-01-28

**Authors:** Ani S. Movsisyan, Kiumarss Nasseri, Theresa H. Keegan

**Affiliations:** 1 Department of Public Health Sciences, University of California Davis, Davis, CA USA; 2 UC Davis Comprehensive Cancer Center, University of California Davis Medical Center, Sacramento, CA USA; 3 International Health and Epidemiology Research Center, Sherman Oaks, USA

**Keywords:** Armenian, Ethnicity, Surname List, Middle East, Identification

## Abstract

**Background:**

There are an estimated 460,000 Armenians in the United States, and more than half live in California. Armenian-Americans are generally represented within the ‘White’ or ‘Some Other Race’ race categories in population-based research studies. While Armenians have been included in studies focused on Middle-Eastern populations, there are no studies focused exclusively on Armenians due to a lack of standardized collection of Armenian ethnicity in the United States or an Armenian surname list. To fill this research gap, we sought to construct and evaluate an Armenian Surname List (ASL) for use as an identification tool in public health and epidemiological research studies focused on Armenian populations.

**Methods:**

Data sources for the ASL included the California Public Use Death Files (CPUDF) and the Middle Eastern Surname List (MESL). For evaluation of the ASL, the California Cancer Registry (CCR) database was queried for surnames with birthplace in Armenia and identified by the MESL.

**Results:**

There are a total of 3,428 surnames in the ASL. Nearly half (1,678) of surnames in the ASL were not identified by the MESL. The ASL captured 310 additional Armenian surnames in the CCR than the MESL.

**Conclusions:**

The ASL is the first surname list for identifying Armenians in major databases for epidemiological research.

**Supplementary Information:**

The online version contains supplementary material available at 10.1186/s12874-023-01848-1.

## Background

US Census race/ethnicity categories provide critical information allowing for health disparities to be recognized and addressed [1]. These categories have lacked details to capture existing health disparities affecting specific communities, including Armenian-Americans [1]. According to the 2019 US Census American Community Survey, the premier source for population information in the United States, there are 458,364 people of Armenian ancestry living in the United States, and 42% (191,252) are foreign born [2]. More than half (245,774) of all Armenians in the United States reside in California [2]. Armenian-Americans are officially included within the ‘White’ or ‘Some Other Race’ race categories in population-based research studies [3], while genetic, and cultural factors are likely to underlie different health patterns among Armenians compared with other race/ethnic groups [4, 5]. For example, while health-related research among Armenians is limited in the United States, a prospective cohort study of Armenian and non-Armenian patients in a county hospital based in Los Angeles, California, showed that genetic, cultural, and dietary factors may contribute to Armenian ethnicity being associated with cardiovascular disease risk [6].

Using a common surname list is often employed to identify and study various ethnic groups [7–9]. In particular, surname lists have been used to study cancer patterns among Arab-American and Middle-Eastern populations in California [7, 9–11]. Studies using the Middle-Eastern Surname List (MESL), which included Armenians, showed notably different cancer patterns between the Middle-Eastern and non-Hispanic, non-Middle-Eastern White populations [9]. While Armenia is geographically proximal to Middle-Eastern countries and some cultural and social traditions are shared, similar adverse cancer patterns are not observed among its closest neighboring countries, including Turkey, Georgia, and Azerbaijan [4]. Other studies on cancer among Middle-Eastern populations in California included Armenians within a broad Middle-Eastern race/ethnic group, but the challenge of identifying Armenians as a separate group has precluded studies specific to the Armenian population [9–12]. A surname list specific to Armenians will allow for the study of cancer patterns among this population, and can serve as a valuable tool for studying the Armenian population in various epidemiological subfields [13].

While the inclusion of Armenians in the Middle-Eastern studies is certainly an important step towards better representation of Armenians in research, studies specifically on Armenians are necessary to better understand health patterns among Armenians around the world [9–12]. We sought to bridge this gap by creating and evaluating an Armenian Surname List (ASL) to identify Armenians in large population-based research databases. We created the ASL by linking an extract of Armenian surnames from the MESL to the California Public Use Death Files (CPUDF) and we used birthplace in Armenia to identify additional Armenian surnames. We evaluated the ASL by comparing with a population-based cancer registry database containing Armenian surnames and birthplace and using a name checking technology to rank the country of origin of the surnames in the ASL.

## Methods

### Data sources

#### California public use death files

The CPUDF is available through the Center for Health Statistics and Informatics (CHSI) branch of the California Department of Public Health (CDPH) and contains information about in-state California deaths [14]. An annual file is released approximately four months after the end of the calendar year. The CPUDF from 1905–2020 contains the following information: first name, middle name, last name, sex, date of birth, place of birth, place of death, date of death, and father’s last name. Father’s last name was available from 1940–2020. Using SAS version 9.4 (SAS Institute, Inc.), individual year death files from 1905–2020 were consolidated into one large file. The consolidated CPUDF from 1905–2020 contained a total of 16,949,541 records and 1,121,710 unique surnames.

#### Middle Eastern Surname List (MESL) Armenian surname extract

The MESL was created in 2007 and was extracted from a NUMIDENT extract file, a data source provided by the US Social Security Administration that began collecting place of birth in 1979 [9]. The Arab surname list was also used as a source of additional Middle-Eastern surnames [7]. The MESL contained 47,574 surnames [9]. Based on birthplace in Armenia, 1,332 surnames were extracted from the MESL and used in this project. Surnames in the MESL are truncated at 10 digits. As such, the extract file contains some truncated Armenian surnames [9].

#### California cancer registry

The California Cancer Registry (CCR) is a comprehensive state-wide cancer surveillance program and meets SEER standards for quality and completeness of data [15]. The CCR is also Gold Certified by the North American Association of Central Cancer Registries (NAACCR) and is one of the largest cancer registries in the world [15]. The CCR research file provides patient demographics, country of birth, primary tumor site, tumor morphology and stage at diagnosis, first course of treatment, and follow-up for vital status on all cancers except for non-melanoma skin cancers [12]. We used the CCR research file with cancer diagnoses from 1988 to 2021. We used patient last name, maiden name, and father’s surname when available to create the list of CCR surnames. Because an estimated 98% of people born in Armenia are of Armenian ancestry, birthplace in Armenia is a relatively reliable measure for Armenian ancestry [16]. Therefore, we used the country of birth variable in the CCR to retrieve all surnames with birth in Armenia. In the CCR, there is a variable indicating whether a patient’s last name is on the MESL.

### Analyses

#### Construction of the Armenian surname list

##### Step 1: California Public Use Death Files (CPUDF) and Armenian surnames from Middle Eastern Surname List (MESL) probabilistic linkage

We used Match*Pro to link surnames in the CPUDF with Armenian surnames from the MESL. Match*Pro is a probabilistic linkage software developed by IMS (Information Management Services, Inc.) under contract with the National Cancer Institute (NCI). The Match*Pro configuration page includes blocking and matching methods, adjusting for blocking sensitivity, and manual review tools to filter categorized matches. Last Name was the comparator of choice and the blocking strategy selected was *Soundex*, which is a phonetic algorithm for indexing names based on sound rather than spelling [17]. The *Soundex* feature allowed for truncated Armenian last names in the MESL to be included in record comparisons. It also allowed flexibility in the matching process for surnames with special characters including spaces, hyphens, and capitalizations. For example, the surname ‘Ter-Minasyan’ was scored as a perfect match with ‘Ter Minasyan’.

All linked records were manually reviewed using the Match*Pro Linkage Results interface. In this step, records were reviewed to sort matches at the surname level. Linkage results were manually separated into two categories of match or non-match described in Supplemental Table 1. This linkage was not limited by birthplace, allowing for the identification of Armenian surnames with birth outside of Armenia.

##### Step 2: Selection of surnames by birthplace in Armenia

Surnames from the CPUDF that were born in Armenia, but did not link with existing Armenian surnames in the MESL (Step 1), were manually reviewed and surnames meeting the following two criteria were deleted from the list: 1) had a length of less than 5 characters or 2) did not have a common Armenian surname suffix *(ian, yan, ians, yans, iants, yants)*, a common Russian patronymic suffix (*ov, ova)* [18, 19], and were less than 12 characters long. These deletions were necessary to remove common non-Armenian surnames such as ‘Smith’, ‘Abad’, ‘Ryan’, and ‘Weatherford’, and to retain Armenian surnames with less common suffixes and at least 12 characters long such as ‘Hambartsum*anz*’ and ‘Ter-Prakhour*any*’. Because many Armenians changed the suffix of their last names during the USSR period to follow Russian patronymic suffixes, we included Armenian last names with a common Russian suffix. All of the 1,332 unique Armenian surnames from the MESL that were not identified by the CPUDF/MESL Armenian surnames linkage nor by the birthplace in Armenia queries in the CPUDF were retained in the ASL.

#### Evaluation of the Armenian surname list

##### Step 1: Compare Exact Surnames between the ASL and California Cancer Registry (CCR) list of surnames with known birthplace in Armenia

Since birthplace in Armenia is a reliable measure for Armenian ancestry [16], we compared the ASL with the CCR Birthplace in Armenia surname list to evaluate the performance of the ASL in identifying Armenian surnames in large research databases. To compare the ASL with the CCR Birthplace in Armenia list, we calculated the number of surnames that were identified in both versus only one of the lists. We presented results in a Venn diagram, with counts representing unique surnames. We calculated the proportion of surnames in the CCR with birthplace in Armenia that were in the MESL. We also compared the proportion of surnames in the CCR Birthplace in Armenia List that were identified by the MESL and the ASL.

##### Step 2: NamSor country of origin ranking of ASL and CCR Birthplace in Armenia list

We also sought evaluate the country of origin of surnames in the ASL compared to the CCR Birthplace in Armenia list because surnames with birthplace in Armenia are likely to have originated in Armenia and therefore represent Armenian ethnic identity [16, 20]. We used an independent onomastic classification tool, NamSor, to compare the country of origin rank of surnames in each list. NamSor is a name checking technology that uses applied onomastics to classify names by gender, country of origin, ethnicity, and diaspora [21–23]. Naïve Bayes Classifiers are a class of algorithms used by NamSor for ranking and classification purposes [23]. The NamSor Country of Origin feature returns a list of the top 10 countries of origin, ranked from most likely to least likely. We compared the country of origin of surnames in the ASL to the CCR Birthplace in Armenia list.

## Results

### Construction of the Armenian surname list

**Fig. 1:**
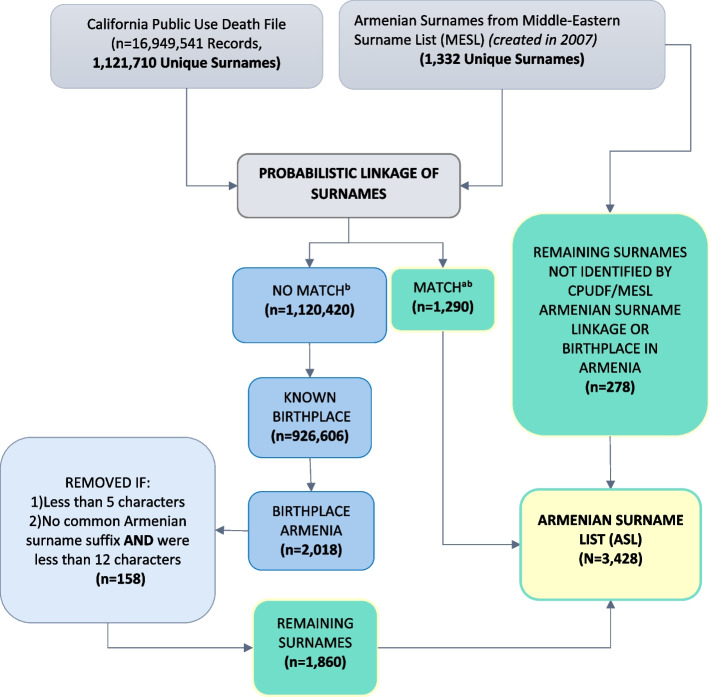
Flowchart of the Armenian Surname List Construction Process. ^a^Soundex Blocking Strategy was used in Match*Pro Linkage Software, allowed for identification and addition of surnames with different spellings of the 1,332 MESL Armenian surnames [17]. ^b^Refer to Supplemental Table 1 for Manual Review Categorization Rules

After manual review of linked surnames between the existing Armenian surnames in the MESL and the CPUDF, we selected 1,290 unique surnames to include in the ASL (Supplemental Table 1, Fig. 1).

The Soundex blocking strategy allowed for truncated surnames in the MESL to match with full Armenian surnames in the CPUDF. For example, the surnames ‘Haroutunia’ and ‘Martirosya’ from the MESL matched with the surnames ‘Haroutunia**n**’ and ‘Martirosya**n**’, respectively, from the CPUDF. Soundex also allowed for flexibility in the spelling of last names and surnames with different spellings were included in the ASL as unique surnames. For example, the surname ‘Mahtes**yan**’ from the MESL matched with the surname ‘Mahtes**ian**’ from the CPUDF and both surnames were included in the ASL. The Soundex blocking strategy allowed for the identification of 232 (17.9%) unique surnames from this linkage that would not have been identified by an exact matching strategy.

There were an additional 2,018 unique surnames in the CPUDF with a birthplace of Armenia that were not identified by the Armenian surnames in the MESL (Table 1). After manually reviewing these surnames, 158 were removed, and the remaining 1,860 surnames were included in the ASL (Fig. 1). From the existing list of 1,332 Armenian surnames in the MESL, 278 surnames did not appear in the CPUDF and were retained in the ASL, including ‘Ter-Galoustian’, ’Ambartsumi’, ‘Mikhailov’, ‘Nerses’, and ‘Oganesov’. Because we retained all Armenian surnames from the MESL, only those surnames from the MESL were truncated at 10 digits in the ASL. The final ASL has a total of 3,428 unique surnames.

**Table 1:** Number and Percentage of Surnames Identified by the Middle Eastern Surname List (MESL)^a^ via Probabilistic Linkage with California Public Use Death File, 1905–2020

	**Birthplace in Armenia in the California Public Use Death File**			
**Identified by MESL** **Armenian Surnames**	Yes	No	Unknown	Total
Yes	720 (26.30)	485 (0.05)	85 (0.04)	1,290 (0.11)
No	2,018 (73.70)	924,679 (99.95)	193,819 (99.96)	1,120,516 (99.89)
Total	2,738 (100)	925,164 (100)	193,904 (100)	1,121,806 (100)

^a^List of Armenian Surnames from the MESL created in 2007

### Evaluation of the Armenian surname list

**Fig. 2:**
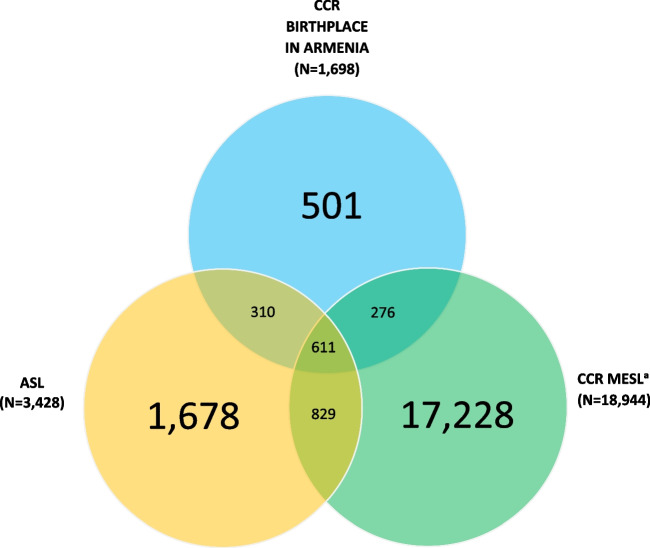
Counts of Overlapping Surnames between the Armenian Surname List (ASL), California Cancer Registry (CCR) Birthplace in Armenia List, and Middle Eastern Surname List (MESL) from the CCR. ^a^Includes all surnames with known country of birth in the California Cancer Registry that indicate surname is on the MESL created in 2007

There were 1,698 unique surnames identified by the CCR with birthplace in Armenia. Of the surnames in the CCR Birthplace in Armenia Surname List, 921 (54.2%) were also identified by the ASL (Fig. 2). There were 18,944 surnames in the CCR file with a known birthplace country that had MESL surnames. Out of 921 surnames, the ASL identified 310 (33.7%) additional surnames in the CCR Birthplace in Armenia list than the MESL. Of the 3,428 surnames in the ASL, 1,678 (48.9%) were not identified in the CCR Birthplace in Armenia list nor the MESL.

**Fig. 3:**
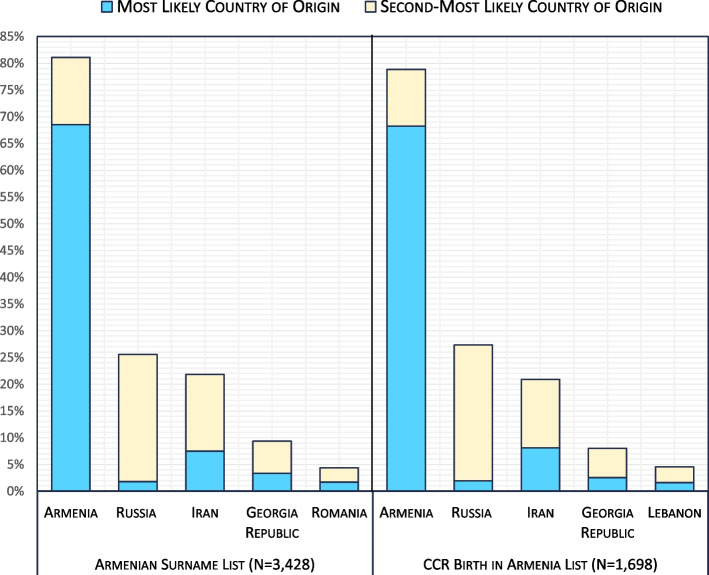
Percentage of Surnames in the Armenian Surname List and California Cancer Registry Birthplace in Armenia List Ranked by Most Likely Country of Origin and Second-Most Likely Country of Origin. The *Country of Origin* feature in NamSor was used to rank country of origin for each surname and returned a list of the top 10 countries of origin ordered from most likely to least likely [21, 22]. Only the top five countries of origin ordered by combined percentage of most likely and second most-likely country of origin are presented for each list

Of the 3,428 surnames in the ASL, 2,349 (68.5%) had Armenia ranked first as the most likely country of origin and 431 (12.6%) as the second-most likely country of origin from NamSor (Fig. 3). There were 257 (7.5%) with Iran listed first, 114 (3.3%) with Georgia Republic listed first, 63 (1.8%) with Russia listed first, and 59 (1.7%) with Romania listed first. Of the 1,698 surnames in the CCR Birthplace in Armenia list, 1,159 (68.3%) had Armenia ranked first as the most likely country of origin from NamSor (Fig. 3). Iran followed with 138 (8.1%) of surnames, then Georgia Republic with 43 surnames (2.5%), Russia with 33 (1.9%) surnames, and Lebanon with 27 (1.6%) surnames.

## Discussion

To our knowledge, this is the first study to create and evaluate a specific Armenian surname list for research purposes. Prior work in Middle Eastern populations highlighted the need for future research to refine procedures pertaining to ethnic overlaps in the MESL [9]. The ASL is a significant step towards increasing clarity and understanding of public health patterns in Armenians. The ASL builds upon Armenian surnames in the MESL by additionally including surnames from over a hundred years of death records in California.

Access to death records in California to create the ASL is particularly advantageous because Armenian immigration to the state dates back to the 1800s and California remains the state with the largest population of Armenians in the United States [24]. The utilization of the Soundex blocking strategy in the linkage between the MESL Armenian surnames and the CPUDF allowed for the identification of nearly 18% of the surnames added to the ASL. As such, when the ASL is used in probabilistic linkages with large databases, we recommend selecting Soundex as a blocking strategy during the linkage configuration process to maximize the number of Armenians identified [17].

During the process of creating the ASL, we considered historical and political events that may have impacted data collection and representation of countries of birth. Specifically, Armenia gained its independence from the Union of Soviet Socialist Republics (USSR) in 1991, raising the question of whether surnames of those born in Armenia during the USSR (1920–1991) had Armenia listed as their country of birth [25]. Our analyses showed that approximately 95% of records in the California death files with birthplace in Armenia were born between 1920–1990, suggesting that surnames of people born in Armenia during the USSR were successfully identified and included in the ASL. A common challenge faced by researchers during the creation of surname lists is the changes of surnames over time. Because Russian was the official language of the USSR, individual countries within the USSR developed onomastic systems that followed Russian naming conventions [26]. Since Armenia was under USSR rule for the majority of the twentieth century, we included Armenian surnames with common Russian suffixes -*ov* and -*ova *in the ASL, consistent with the MESL [19, 27]. Out of the 3,428 unique surnames in the ASL, 73 (2%) have a suffix of -*ov* or -*ova* and 70 (96%) of these surnames had a birthplace of Armenia in the CPUDF. Given the unique characteristics of Armenian surnames and the small percentage of surnames in the ASL with a -*ov*, -*ova *suffix, we do not expect a remarkable number of non-Armenian Russians to be identified by the ASL [18, 19].

Another challenge in creating surnames lists is changing surnames due to marriage or to follow naming conventions of a new culture and society after immigration. To address this issue, we included all available father’s last names from the California death records for both males and females. For example, father’s last name of ‘Daniel**ian**’ was identified with the same record where the last name followed a different naming convention of ‘Daniel**son**’. A common limitation we faced is the lack of mother’s last name, which may have precluded the identification of children of Armenian mothers with an Armenian last name and fathers with non-Armenian last names.

Using full surnames, as we did when creating the ASL, is particularly advantageous in large database linkages for several reasons. In probabilistic linkages, full surnames will have a higher similarity score when they match a full versus truncated version of the surname. Also, deterministic linkages can be utilized with non-truncated surnames. Lastly, name-checking technologies, such as NamSor, will return more accurate rankings of surname country of origin with full surnames, as many naming conventions, including Armenian, rely on the suffix to identify ethnic origin. Due to the novelty of this surname list, we did not have a ‘gold standard’ or contact people to confirm their ethnic origin to validate the ASL. While previous researchers who have created surname lists, such as the Arab surname list, have used self-reported ethnicity from telephone surveys as a ‘gold standard’ when validating new surname lists [28], we did not have self-reported ethnicity for the purposes of this study. Therefore, we compared overlap of surnames between the CCR and the ASL.

Comparisons of the ASL with a list of MESL surnames from the CCR were suggestive that the ASL identifies a considerable percentage (34%) of Armenian surnames not identified by the MESL. This may relate to a few characteristics of the list of Armenian surnames extracted from the MESL. In addition, the Armenian surnames were extracted from NUMIDENT using a filter of birth in Armenia and may exclude surnames of people who were born in other countries and who had different surname conventions than those who were born in Armenia. For example, in the CPUDF and MESL Armenian surname linkage results, we observed that 83% of surnames with birthplace in Russia followed a surname convention of the suffix beginning with the letter *I* (*ian, ians, iants*), while 22% of surnames with birthplace in Armenia had surname suffixes of (*ian, ians, iants*) and 77% had surname suffixes beginning with the letter *Y* (*yan, yans, yants*). We attempted to address this within the ASL, as the surnames added to the ASL were not limited to birth in Armenia. A potential limitation of the ASL is that a small percentage of people born in Armenia may have a non-Armenian last name or may not have Armenian origin because we retained Armenian surnames from the MESL. Further research application of the ASL will clarify whether any enhancements to the ASL are warranted.

Country of origin checks using NamSor for the ASL surnames and the CCR Birthplace in Armenia surnames retrieved similar results, with Armenia ranked as the most likely or second-most likely country of origin for more than three-fourths of surnames, suggesting that the ability of ASL to identify Armenian surnames is comparable to the ability of a list containing surnames identified solely by birthplace in Armenia. Because the two lists had only about 30% overlap between surnames, it may be worthwhile to consider the combination of the two lists to optimize the identification of Armenian surnames in large population-based databases.

The use of the ASL can increase the representation of Armenians in public health research. For example, the ASL can be used to identify Armenians in population-based cancer registry databases and observe cancer patterns by demographic and clinical factors. This will result in a clearer representation and understanding of cancer patterns among racial/ethnic categories where Armenians are commonly included, such as Middle-Eastern and White. Such data can inform health education and awareness campaigns to increase access to preventive healthcare services, such as screening, in the Armenian population. The ASL allows for the identification and representation of Armenians in a myriad of research endeavors and lays the groundwork as a valuable resource for research studies in public health, epidemiology, and other fields concerning Armenian communities.

## Supplementary Information

**Additional file 1: Supplemental Table 1.** Manual Review Categorization Rules, Public Use Death Files 1905-2020 and Middle Eastern Surname List (MESL) Armenian Surname Probabilistic Linkage.

## Data Availability

The data that support the findings of this study are available from the California Department of Public Health and the California Cancer Registry. Access is granted through an application process by the management or data custodians (https://www.cdph.ca.gov/Programs/CHSI/Pages/Data-Applications.aspx) and (https://www.ccrcal.org/retrieve-data/).
