# Supplementary material for: Construction of the Armenian Surname List (ASL) for public health research
Source: BMC Med Res Methodol. 2023 Jan 28;23:29. doi: 10.1186/s12874-023-01848-1 (PMC9883902; doi:10.1186/s12874-023-01848-1)
Supplement: Supplementary file 1 — Additional file 1: Supplemental Table 1. Manual Review Categorization Rules, Public Use Death Files 1905-2020 and Middle Eastern Surname List (MESL) Armenian Surname Probabilistic Linkage. [file 12874_2023_1848_MOESM1_ESM.docx]

**Supplemental Table 1. Manual Review Categorization Rules, Public Use Death Files 1905-2020 and Middle Eastern Surname List (MESL) Armenian Surname Probabilistic Linkageᵇ Results.**

| **RULE** | | **N Records** | **%** | **SIMILARITYᵃ** |
| --- | --- | --- | --- | --- |
| MATCH | | | | |
| 1 | Exact Match | 31,176 | 92.72 | 1.00 |
| 2 | Spelling variation or truncation **AND** 'yan','ian','ians', ‘yans’,’iants’,'yants', ending convention | 2,135 | 6.35 | 0.87-0.98 |
| 3 | Spelling variation or truncation **AND** ≥10 digits | 30 | 0.09 | 0.88-0.98 |
| NON MATCH | | | | |
| 4 | Did not meet any match categorization rule | 282 | 0.84 | 0.87-0.98 |
|  | **Total** | **33,623** | **100** |  |

ᵃBased on matching and blocking threshold settings selected on the linkage configuration page, the Match*Pro results file provides a similarity measure from 0-1 for each linked pair.

ᵇMatch*Pro Probabilistic Linkage Configuration: On the Matching tab, the last name and father’s last name variables from the CPUDF were selected as the File 1 Field, and the last name from the list of Armenian surnames from the MESL was selected as the File 2 Field. On the Blocking tab, the only blocking field selected was last name.
